# The Association Between Sarcopenia and Stress Urinary Incontinence Among Older Adults in India: A Cross-Sectional Study

**DOI:** 10.7150/ijms.97240

**Published:** 2024-09-03

**Authors:** Bin Zeng, Yuming Jin, Xingyang Su, Mi Yang, Xinyi Huang, Shi Qiu

**Affiliations:** 1 Department of Urology, Institute of Urology and National Clinical Research Center for Geriatrics, West China Hospital, Sichuan University, Chengdu, Sichuan Province, China.; 2 West China School of Public Health and West China Fourth Hospital, Sichuan University, Chengdu, China.; 3 Center of Biomedical Big Data, West China Hospital, Sichuan University, Chengdu, Sichuan, China.

**Keywords:** Sarcopenia, Stress Urinary Incontinence, LASI

## Abstract

**Background:** Older adults in low- and middle-income countries (LMICs) often suffer from both sarcopenia and stress urinary incontinence (SUI), two conditions that can significantly impact their health. However, the relationship between these conditions has not been thoroughly explored.

**Methods:** We conducted a cross-sectional study using data from older adults aged 50 years or older from the first wave of the Longitudinal Ageing Study in India (LASI). Participants with complete data on sarcopenia and SUI were included, excluding female participants who were still menstruating. Sarcopenia was defined as decreased grip strength and slow movement. SUI was assessed based on questionnaire responses about whether the participant had ever passed urine when sneezing, coughing, laughing, or lifting heavy objects. We analyzed the data using multiple logistic regression analysis, interaction tests, and stratified analysis.

**Results:** Our results showed that sarcopenia was positively correlated with SUI in male participants after adjusting for adequate confounding factors (odds ratio [OR] = 1.37, 95% confidence interval [CI] [1.20, 1.56], p < 0.001). This correlation remained stable after adjusting for additional confounding factors (OR = 1.27, 95% CI [1.11, 1.45], p < 0.001). In female participants, a stable correlation between sarcopenia and SUI was also observed after adjusting for appropriate confounding factors (OR = 1.11, 95% CI [1.01, 1.23], p = 0.037). According to the results of interaction tests and stratified analysis, the positive correlation between sarcopenia and SUI is notably stronger among men who abstain from alcohol and women who haven't undergone hysterectomy.

**Conclusions:** Sarcopenia and SUI were positively correlated in older Indian adults, regardless of gender. Drinking and a history of hysterectomy may be important influencing factors for both male and female older adults. Further large-scale clinical trials are necessary to confirm this association.

## Introduction

Sarcopenia, a clinical syndrome characterized by the progressive loss of skeletal muscle mass, strength, and function, has become a significant threat to the health and quality of life of older individuals worldwide. According to data from the Asian Working Group for Sarcopenia (AWGS), the prevalence of sarcopenia ranges from 5.5% to 25.7%, with males being more affected (5.1% - 21.0% for males vs. 4.1% - 16.3% for females)^1^, ^2^. In India, approximately 17.5% of the population suffers from muscle loss, a rate significantly higher than that in other Asian countries and Europe^3^. Among the many reported risk factors for sarcopenia, age may be the most significant^4^. However, family status, lifestyle, and malnutrition are also independently associated with sarcopenia^5^. Additionally, sarcopenia can lead to complications from various diseases in the older population, such as stroke, cancer, and Parkinson's disease^6^. Thus, focusing on sarcopenia in older individuals is crucial for improving their quality of life. Stress urinary incontinence (SUI) is a common condition among the elderly, characterized by involuntary urine leakage from the external urethral orifice when abdominal pressure increases, such as during sneezing or coughing. The prevalence of SUI is reported to range from 29% to 75%, with an average of 48%, depending on age^7^. SUI usually leads to a serious deterioration of the quality of life of the older and causes huge costs to patients and the health care system^8^. Although SUI is more prevalent among older women, a significant proportion of older men also experience it. Reports indicate that the overall prevalence of urinary incontinence in men ranges from 12.7% to 17%, with 24.5% of these cases being attributed to SUI^9^. Therefore, SUI is a significant concern for the older population, regardless of gender.

It has been proposed that the onset of SUI is primarily related to pelvic floor weakness, loss of detrusor contraction, or sphincter control dysfunction^10^. Sarcopenia is characterized by progressive and systemic loss of skeletal muscle quality, strength, and function^11^. Therefore, we naturally connected the two. To our knowledge, only a few studies have explored the correlation between sarcopenia and urinary incontinence, particularly in the older male population^12^. To better understand the relationship between sarcopenia and SUI among the older, particularly in developing countries, we analyzed the data from the Indian Longitudinal Aging Study (LASI).

## Methods

### Data and participants

The data used in this study was obtained from the first wave of Indian Longitudinal Aging Study (LASI) conducted in 2017-2018. This is the first study on aging in India, which focuses on the health, economic, social, and psychological aspects of the aging process in India. The first wave of the survey adopted a multi-stage stratified regional probability cluster sampling design to select participants. Finally, 72,262 people over 45 years old and their spouses were included in the survey.

**Figure 1:**
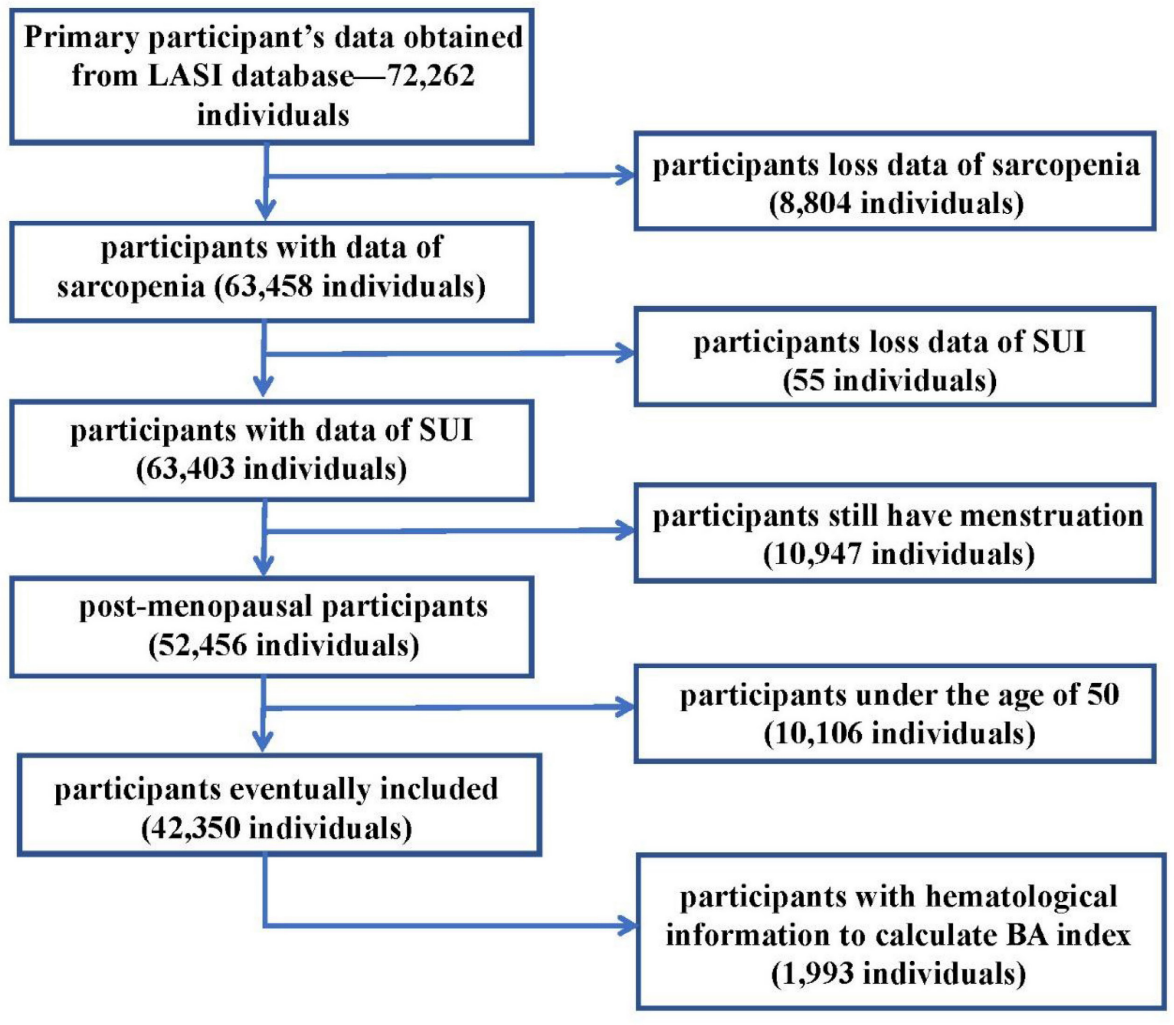
** Flow chart of inclusion and exclusion criteria for our study. Notes:** LASI: Longitudinal Ageing Study in India; SUI: Stress Urinary Incontinence; BA index: Biological Aging index.

Previous literature indicates minimal variations in muscle mass and strength among individuals aged 30 to 50, but a noticeable acceleration in muscle decline is observed after the age of 50^13^. Thus, in this article, we included Indian adults aged 50 years or older with data of sarcopenia and SUI. Female participants aged 50 years or older who were still menstruating were excluded. The remaining 42,350 participants who met the requirements for admission and discharge were finally enrolled in the study. In the population meeting the criteria for inclusion, there were 1,993 people who have laboratory examination information in the LASI-DAD database. In view of the role of aging in SUI proposed in the EAU Guide^14^, we introduced the BA (Biological Aging) index into this article. In this paper, the Biological Age (BA) index was calculated using the method proposed by Klemera and Doubal, based on 8 biomarkers^15^, ^16^. And we used the hematological information of these people to calculate the BA index. The participant selection process was shown in the Figure 1. Each participant has signed a relevant written informed consent form. The details can be found on the LASI wave 1 website^17^.

### Sarcopenia and stress urinary incontinence

In this article, sarcopenia was defined according to the criteria of the Asian Working Group for Sarcopenia: participants were classified as having sarcopenia if they exhibited decreased grip strength (less than 28 kg for men and less than 18 kg for women) and slow movement (a 6-meter walk test result of less than 1.0 m/s)^6^. Grip strength (kg) was measured using a dynamometer. All participants followed a standard procedure: they squeezed the handgrip machine as hard as possible three times, with 60-second intervals between each measurement. The maximum value recorded was taken as the final grip strength of the participants^18^. Moving speed was assessed by timing the duration required to walk 6 meters at a normal pace, without slowing down. The average of at least two trials was used as the recorded speed^19^.

The incidence of SUI was determined through a questionnaire survey. According to the EAU guidelines and relevant literature, SUI is defined as involuntary urine leakage from the external urethral orifice when abdominal pressure increases, such as during sneezing or coughing^14^, ^20^. Therefore, in this article, we evaluated SUI based on questionnaire data, specifically assessing whether respondents had ever experienced involuntary urine leakage when sneezing, coughing, laughing, or lifting heavy objects 21.

### Covariates

The demographic characteristics of participants at baseline were extracted from the LASI database and included age, BMI, marital status, education level, number of Combined Chronic Diseases (CCDs), and history of drinking and smoking. Marital status was categorized as married or partnered, widowed, and other. Education was classified as no formal education, middle school or less, secondary, and higher secondary or above. CCDs were defined as having one or more chronic diseases such as hypertension, diabetes, heart disease, or stroke. Drinking and smoking history were categorized based on habits (never, current, or past). The BA index in this paper was calculated using the formula: BA index = (BA - age) / age^22^. We also extracted additional potential confounding factors from the database, including place of residence, caste, religious belief, and levels of physical activity, as well as assistance needed for Activities of Daily Living (ADL) and Instrumental Activities of Daily Living (IADL). We included hysterectomy history (for female participants), economic status, and waist-hip ratio. In this study, place of residence was categorized as either village or city. For caste, reflecting India's unique ethnic system, we classified participants into scheduled castes, scheduled tribes, other backward classes, and non-scheduled or other castes. Religious belief was categorized into Muslims, Christians, Hindus, and others based on mainstream religious and cultural affiliations in India. Physical activity was assessed by asking participants how often they engaged in mild or vigorous activities, such as chopping firewood, lifting heavy objects, cleaning the house, washing clothes, or fetching water. The frequency was classified as less than once a week. We recorded the number of participants needing care due to ADL/IADL impairments. Hysterectomy history referred to whether a female participant had ever undergone a hysterectomy. Economic status was evaluated based on annual per capita consumption expenditure from household consumption data.

### Statistical analysis

In this study, continuous variables of baseline characteristics were reported as means and standard deviations, while categorical variables were expressed as ratios and percentages. For continuous variables, the Kruskal-Wallis rank sum test was used to determine P-values. For categorical variables with theoretical frequencies less than 10, Fisher's exact test was applied. To account for the influence of various confounding factors, we adjusted for different covariates and employed multivariate logistic regression analysis to examine the relationship between sarcopenia and SUI. Given that the mechanisms of SUI differ between men and women, we performed separate univariate logistic regression analyses for each gender^23^. First, we adjusted no variables for regression analysis as Crude Model. Then, the first model was adjusted for age, education, marriage, Annual per capita consumption expenditure, live in a village or a city, caste and religion (Model I); The second model adjusted for age, education, marriage, live in a village or a city, Annual per capita consumption expenditure, number of CCDs recoded, BMI, Waist-hip ratio, drinking, smoking, vigorous physical activity, mild physical activity, religion and caste (Model II). For female participants, we adjusted the confounding factor "hysterectomy history" in addition to the above confounding factors in model II. In order to further verify the reliability of these correlations, we conducted interaction tests and stratified analysis on the relevant variables. Similarly, due to the differences between men and women in the mechanisms of SUI, we performed interaction tests and stratified analysis on men and women respectively. Among male participants, we conducted interaction tests and stratified analysis on the following variables: drinking, smoking, BA index (categorized as: no or premature senility), age (classified as: <=60; > 60, <=70 and >70), care for ADL/IADL (yes or no), BMI (It is divided into <30 and ≥ 30 according to obesity), mild or vigorous physical activity (less than once a week or more). In female participants, hysterectomy, drinking, mild or vigorous physical activity, BMI, age, BA index, smoking, and care for ADL/IADL were performed the interaction tests and stratified analysis. The variables adjusted by different models are the same as those performed in multivariate logistic regression analysis. All statistical results of this article were performed by R 4.1.2 (R project), and p<0.05 was considered statistically significant.

## Results

### Baseline characteristics

The study included 42,350 participants, with 14,282 classified as having sarcopenia and 28,068 having normal grip strength and movement pace. The average age of the participants was 62.63 ± 8.98 years, with 49.35% female and 50.65% male. The average BA index for the entire cohort was 0.0020 ± 0.1095, while for the sarcopenia group, it was 0.0126 ± 0.1123. Among the participants, 82.43% had received no more than a middle school education, 73.75% were married or had a partner, and 6.73% were classified as obese. Of the total participants, 34,235 had never consumed alcohol, and 79.55% had never smoked. Baseline characteristics of the participants are detailed in Table 1. Overall, participants with sarcopenia were more likely to be older, have lower levels of education, possess a higher BA index, and have more chronic diseases. A total of 3,474 participants were defined as having SUI, with those having sarcopenia showing a significantly higher proportion of SUI compared to those with normal muscle strength and movement pace (10.97% vs. 6.79%). Detailed information is presented in Supplementary Table 1.

### Association between sarcopenia and stress urinary incontinence

Table 2 presents the logistic regression analysis results of the association between sarcopenia and SUI symptoms for male (Table 2A) and female participants (Table 2B). The results indicate a positive correlation between sarcopenia and SUI in both males (OR = 2.13, 95% CI [1.89, 2.39], p < 0.001) and females (OR = 1.40, 95% CI [1.28, 1.53], p < 0.001). Among male participants, after adjusting for age, education, marital status, place of residence (village or city), caste, religion, and annual per capita consumption expenditure, Model I showed an OR of 1.37 (95% CI [1.20, 1.56], p < 0.001). This association remained significant even after adjusting for all covariates in Model II (OR = 1.27, 95% CI [1.11, 1.45], p < 0.001). Similarly, in female participants, after adjusting for relevant confounding factors, the positive correlation between sarcopenia and SUI remained stable, with a significant relationship still observed (OR = 1.11, 95% CI [1.01, 1.23], p = 0.037). These regression analysis results indicate that sarcopenia is positively associated with SUI across both genders.

### Interaction tests and stratified analysis

**Figure 2:**
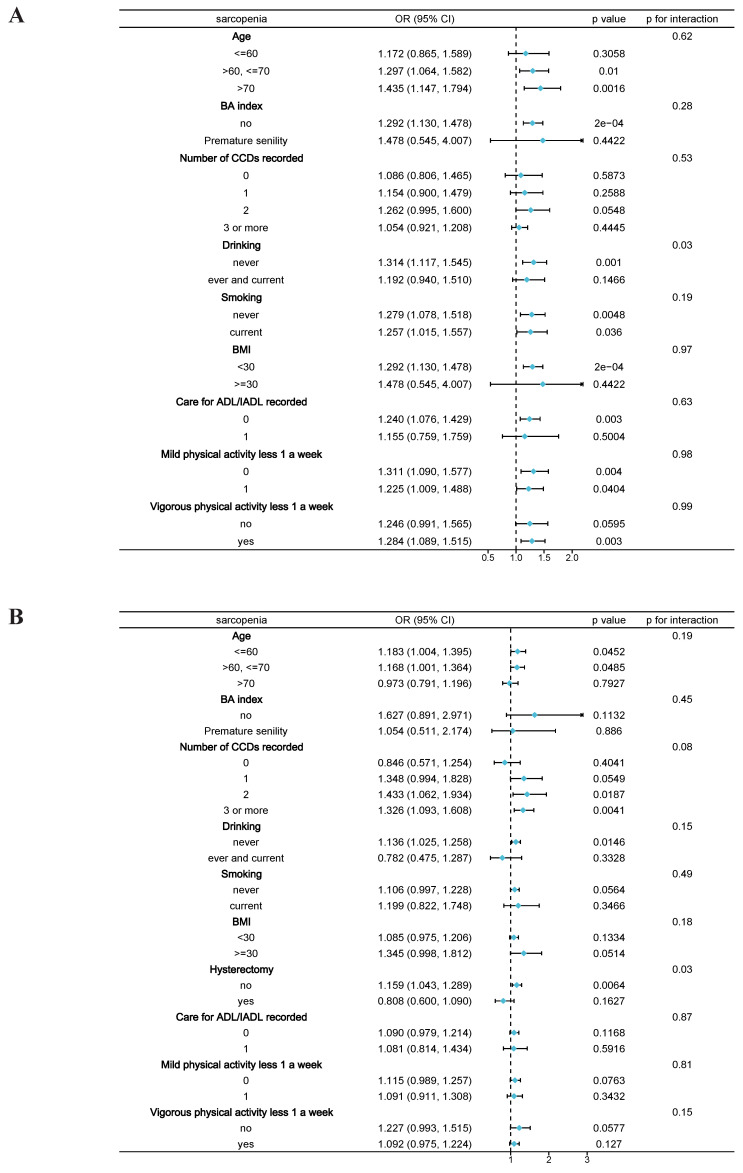
** Subgroup analysis between sarcopenia and stress urinary incontinence. A,** Male participants; **B,** Female participants. Note: BMI, Body Mass Index; BA index, Biological Aging index; CCDs, Combined Chronic Diseases; ADL, Activities of Daily Living; IADL, Instrumental Activities of Daily Living.

Stratified analysis revealed that among the elderly male participants, the presence of sarcopenia was positively associated with SUI (Figure 2A), consistent with the previous results. Subgroup analysis across different variables demonstrated that the positive correlation between sarcopenia and SUI remained stable in older males. Specifically, in elderly males with higher levels of aging (age >70), lower body weight (BMI <30), and those who never drank alcohol, the correlation between sarcopenia and SUI was more pronounced. The interaction test indicated that drinking history might play a significant role in the association between sarcopenia and SUI among male participants (p = 0.028). In elderly female participants, hysterectomy history showed a significant difference (p = 0.031), while the interaction tests for other variables did not yield statistically significant results.

## Discussion

In this article, we explored the relationship between sarcopenia and SUI in older adults by analyzing data from the Indian population. To our knowledge, this is the first clinical study in recent years to investigate sarcopenia and SUI among individuals in developing countries. Our regression analysis revealed a positive correlation between sarcopenia and SUI. After adjusting for potential confounding factors such as age, education level, comorbidity, smoking, and drinking history, this positive correlation remained consistent across both genders. The results of interaction tests and stratified analysis showed that among men who abstain from alcohol and women who have not undergone hysterectomy, the association between sarcopenia and SUI is notably stronger.

We believe that muscle loss may be linked to SUI, as the onset of SUI is primarily associated with pelvic floor weakness, loss of detrusor contraction, or impaired control of sphincter organs^11^. The pelvic floor, including the levator ani muscles, pelvic fascia, and supporting ligaments, forms a crucial supportive structure for the urethra 24. When these structures become weakened due to various mechanisms, they are unable to maintain stability as bladder pressure increases, leading to incontinence^25^. For patients with sarcopenia, the weakening of abdominal muscles and pelvic floor support structures is often involved. Therefore, we suggest a potential relationship between sarcopenia and SUI.

According to our search, only two clinical studies have explored the relationship between sarcopenia and SUI. Erdogan et al.^12^ took 802 older women over 60 years old from Turkey as the research object to explore the relationship between sarcopenia and urinary incontinence. Their results suggested that UI is independently related to sarcopenia in the older female population when muscle mass is adjusted by weight and is also independently related to the existence of low muscle mass when muscle mass is adjusted by weight or BMI. Nevertheless, in this article, we did not observe the important role of BMI in the relationship between sarcopenia and SUI, no matter in interaction test or stratified analysis. In our study, we believe that the reason why BMI has not become a significant influencing factor for the relationship between sarcopenia and SUI may be that in India, a developing country, there is a huge difference in the number of obese and non-obese people (2,846 vs 39,445). Another clinical study^26^, explored the relationship between the type of urinary incontinence, grip strength and pelvic floor muscle strength in adult women based on the data of 92 women who reported symptoms of urinary incontinence. Their results indicated no direct correlation between the type of urinary incontinence and grip strength. However, there was a positive correlation between pelvic floor muscle strength and grip strength (p = 0.045, r = 0.298), suggesting that low grip strength may be indicative of weak pelvic floor muscles. This association between sarcopenia and urinary incontinence could be explained by this finding, which aligns with our results. We observed a positive correlation between sarcopenia and SUI by analyzing data from 42,350 older adults in India. While their study did not directly explore the relationship between sarcopenia and urinary incontinence, it focused on the relationship between grip strength and urinary incontinence. Given the small sample size and the lack of a non-incontinence control group in their research, their findings warrant further validation through more extensive clinical studies.

In this article, the interaction test revealed that alcohol consumption and hysterectomy history may play significant roles in the association between sarcopenia and SUI in older men and older women, respectively. Previous literature presents conflicting views on the role of alcohol in SUI, rendering it a subject of ongoing debate. For instance, two multicenter studies from China have identified alcohol intake as an independent risk factor for women with SUI^27^, ^28^. Women who consumed alcohol had a higher risk of SUI compared to those who did not (OR = 1.22, p = 0.024). However, some literature also indicates that alcohol consumption may not significantly affect the occurrence of SUI^29^. The impact of hysterectomy on SUI has also been widely discussed. A cohort study from Sweden, with a 30-year follow-up published in *The Lancet*, found that women who had undergone hysterectomy had a significantly increased risk of SUI following the surgery^30^. Some scholars believed that hysterectomy may damage the pelvic floor supporting structure and lead to urinary incontinence. After all, pelvic floor structure disorder and weakening is one of the clear causes of SUI found at present^14^. However, in our study, economic and social factors in the Indian population led to significant variations in group sizes. The number of non-drinkers among men is nearly twice that of ex-drinkers and regular drinkers, while the number of women with a history of hysterectomy is only one-eighth of those who have never undergone the procedure. Therefore, further clinical trials are needed to verify the roles of alcohol consumption and hysterectomy in the association between sarcopenia and SUI in elderly men and women.

This study has several notable highlights and advantages. It includes data from 42,350 older adults in India and, to our knowledge, is the first to use LASI national data to explore the relationship between sarcopenia and SUI in the context of developing countries. Our findings reveal a positive correlation between sarcopenia and SUI in the elderly, which may provide insights for other developing nations. Previous literature indicates that the prevalence of sarcopenia among older adult ranges from 9.9% to 40.4%, and the aging rate in low- and middle-income countries (LMICs) is accelerating faster than in high-income countries^31^. It is estimated that by 2050, more than two-thirds of the elderly population will reside in low- and middle-income countries^32^, and sarcopenia may become an increasingly important health problem in low-income and middle-income countries in the next few years^33^. Therefore, it is crucial to focus on the adverse health outcomes of sarcopenia in low- and middle-income countries (LMICs). Our research highlights the importance of addressing health issues such as sarcopenia in developing countries. Unlike previous studies that primarily focused on SUI in older women, our research also includes older men, revealing significant findings in both groups. Additionally, following the EAU guidelines on aging and SUI, we introduced the Biological Age (BA) index in our study. Although no significant effect of the BA index on the relationship between sarcopenia and SUI was observed, it offers a valuable reference for future research on the role of aging in SUI.

Nevertheless, there are several limitations to our research. First, as a cross-sectional study, it cannot establish a causal relationship between sarcopenia and SUI. Second, the definition of SUI is based on participants' self-reports, which may be biased. Due to the stigma associated with urinary incontinence, there could be inaccuracies in the reported data. However, by adhering to the EAU guidelines for defining SUI, we have somewhat ensured the scientific validity of the responses. Third, since SUI is one of the most important complications after prostatectomy for the treatment of prostate diseases, especially prostate cancer, this part of the data was missing in the LASI data, and we believe that this result needs further study for verification. Additionally, the unique cultural customs and social background of the Indian population resulted in significant differences in group sizes within our hierarchical analysis, which could introduce bias. Future research should involve larger and more comprehensive prospective studies in low- and middle-income countries (LMICs) to further validate our findings. Despite these limitations, this study contributes valuable insights into the relationship between sarcopenia and SUI in the older population of LMICs.

## Conclusion

Sarcopenia and stress urinary incontinence (SUI) are prevalent conditions that impact the health of the elderly. Our research indicates a positive correlation between sarcopenia and SUI among older Indians. Therefore, it is crucial to implement appropriate lifestyle interventions to reduce the incidence of sarcopenia and, consequently, the occurrence of SUI symptoms, especially in low- and middle-income countries (LMICs). Further large-scale prospective studies are needed to validate these findings and enhance our understanding of these conditions.

## Supplementary Material

Supplementary table.

## Figures and Tables

**Table 1:** Baseline characteristics of included participants.

Variables	Total	Non-sarcopenia	Sarcopenia	Standardize diff.	P-value
N	42,350	28,068	14,282		
Age (mean ± SD)	62.63 ± 8.98	60.18 ± 7.55	67.45 ± 9.60	0.84 (0.82, 0.86)	<0.001
BA index (mean ± SD)	0.00 ± 0.11	-0.01 ± 0.11	0.01 ± 0.11	0.18 (0.09, 0.27)	<0.001
BMI (mean ± SD)	22.59 ± 4.70	23.03 ± 4.57	21.73 ± 4.85	0.28 (0.25, 0.30)	<0.001
BMI categorical, n (%)				0.05 (0.03, 0.07)	<0.001
<30	23,680 (94.28%)	26,034 (92.83%)	13,411 (94.14%)		
>=30	1,436 (5.72%)	2,011 (7.17%)	835 (5.86%)		
Gender, n (%)				0.13 (0.11, 0.15)	<0.001
male	21,450 (50.65%)	14,815 (52.78%)	6,635 (46.46%)		
female	20,900 (49.35%)	13,253 (47.22%)	7,647 (53.54%)		
Education, n (%)				0.31 (0.29, 0.33)	<0.001
never	20,321 (47.98%)	12,159 (43.32%)	8,162 (57.15%)		
middle school or under	14,591 (34.45%)	10,165 (36.22%)	4,426 (30.99%)		
secondary and higher secondary	5,251 (12.40%)	4,004 (14.27%)	1,247 (8.73%)		
above higher secondary	2,187 (5.16%)	1,740 (6.20%)	447 (3.13%)		
Marriage, n (%)				0.38 (0.36, 0.40)	<0.001
Married or partnered	31,233 (73.75%)	22,253 (79.28%)	8,980 (62.88%)		
Widowed	10,152 (23.97%)	5,172 (18.43%)	4,980 (34.87%)		
Others	965 (2.28%)	643 (2.29%)	322 (2.25%)		
Number of CCDs, n (%)				0.15 (0.13, 0.17)	<0.001
0	8,120 (19.29%)	5,750 (20.59%)	2,370 (16.72%)		
1	9,448 (22.44%)	6,479 (23.20%)	2,969 (20.94%)		
2	8,794 (20.89%)	5,885 (21.08%)	2,909 (20.52%)		
3	15,738 (37.38%)	9,809 (35.13%)	5,929 (41.82%)		
Drinking, n (%)				0.07 (0.05, 0.09)	<0.001
never	34,235 (80.92%)	22,430 (80.00%)	11,805 (82.71%)		
ever and current	8,074 (19.08%)	5,607 (20.00%)	2,467 (17.29%)		
Smoking, n (%)				0.03 (0.01, 0.05)	0.013
never	33,654 (79.55%)	22,204 (79.21%)	11,450 (80.23%)		
ever and current	8,650 (20.45%)	5,829 (20.79%)	2,821 (19.77%)		
Stress Urinary Incontinence, n (%)				0.15 (0.13, 0.17)	<0.001
No	38,876 (91.80%)	26,161 (93.21%)	12,715 (89.03%)		
Yes	3,474 (8.20%)	1,907 (6.79%)	1,567 (10.97%)		

**Note:** BMI, Body Mass Index; BA index, Biological Aging index; CCDs, Combined Chronic Diseases.

**Table 2:** Regression model of the relationship between sarcopenia and stress urinary incontinence.

**A**						
Exposure	Crude Model		Model I		Model II	
	OR (95%CI)	*p*-value	OR (95%CI)	*p*-value	OR (95%CI)	*p*-value
Non-sarcopenia	1		1		1	
Sarcopenia	2.13 (1.89, 2.39)	<0.0001	1.37 (1.20, 1.56)	<0.0001	1.27 (1.11, 1.45)	0.0005
**B**						
Exposure	Crude Model		Model I		Model II	
	OR (95%CI)	*p*-value	OR (95%CI)	*p*-value	OR (95%CI)	*p*-value
Non-sarcopenia	1		1		1	
Sarcopenia	1.40 (1.28, 1.53)	<0.0001	1.13 (1.02, 1.25)	0.0143	1.11 (1.01, 1.23)	0.0365

**A:** for the male participants; **B:** for the female participants Crude Model adjust for: None. **Model I adjust for:** age; education; marriage; Annual per capital consumption expenditure; live in a village or a city; caste; religion. **Model II adjust for:** age; education; marriage; live in a village or a city; Annual per capital consumption expenditure; number of CCDs recorded; BMI; Waist-hip ratio; drinking recorded; smoking recorded; vigorous physical activity less 1 a week; caste; religion; mild physical activity less 1 a week; hysterectomy (only in female participants).

## References

[1] Yoshimura N, Muraki S, Oka H, Iidaka T, Kodama R, Kawaguchi H (2017). Is osteoporosis a predictor for future sarcopenia or vice versa?. Four-year observations between the second and third ROAD study surveys. Osteoporos Int.

[2] Woo J, Leung J, Morley JE (2015). Defining sarcopenia in terms of incident adverse outcomes. J Am Med Dir Assoc.

[3] Tyrovolas S, Koyanagi A, Olaya B, Ayuso-Mateos JL, Miret M, Chatterji S (2016). Factors associated with skeletal muscle mass, sarcopenia, and sarcopenic obesity in older adults: a multi-continent study. J Cachexia Sarcopenia Muscle.

[4] Cruz-Jentoft AJ, Sayer AA (2019). Sarcopenia. Lancet.

[5] Bloom I, Shand C, Cooper C, Robinson S, Baird J (2018). Diet Quality and Sarcopenia in Older Adults: A Systematic Review. Nutrients.

[6] Chen LK, Woo J, Assantachai P, Auyeung TW, Chou MY, Iijima K (2020). Asian Working Group for Sarcopenia: 2019 Consensus Update on Sarcopenia Diagnosis and Treatment. J Am Med Dir Assoc.

[7] Wood LN, Anger JT (2014). Urinary incontinence in women. BMJ: British Medical Journal.

[8] Schiffman M, Lamparello N (2021). Stress Incontinence in Women. N Engl J Med.

[9] Tsakiris P, de la Rosette JJ, Michel MC, Oelke M (2008). Pharmacologic Treatment of Male Stress Urinary Incontinence: Systematic Review of the Literature and Levels of Evidence. European Urology.

[10] Wu JM (2021). Stress Incontinence in Women. N Engl J Med.

[11] Klein G, Hart ML, Brinchmann JE, Rolauffs B, Stenzl A, Sievert K-D (2015). Mesenchymal stromal cells for sphincter regeneration. Advanced Drug Delivery Reviews.

[12] Erdogan T, Bahat G, Kilic C, Kucukdagli P, Oren MM, Erdogan O (2019). The relationship between sarcopenia and urinary incontinence. Eur Geriatr Med.

[13] Keller K, Engelhardt M (2013). Strength and muscle mass loss with aging process. Age and strength loss. Muscles Ligaments Tendons J.

[14] Eau Guidelines on Urinary Incontinence. Presented at the Eau Annual Congress Amsterdam 2020. Isbn 978-94-92671-07-3. Available at:.

[15] Klemera P, Doubal S (2006). A new approach to the concept and computation of biological age. Mech Ageing Dev.

[16] Levine ME (2013). Modeling the rate of senescence: can estimated biological age predict mortality more accurately than chronological age?. J Gerontol A Biol Sci Med Sci.

[17] India Report (2020). Available online at:.

[18] Roberts HC, Denison HJ, Martin HJ, Patel HP, Syddall H, Cooper C (2011). A review of the measurement of grip strength in clinical and epidemiological studies: towards a standardised approach. Age and Ageing.

[19] Arai H, Wakabayashi H, Yoshimura Y, Yamada M, Kim H, Harada A (2018). Chapter 4 Treatment of sarcopenia. Geriatrics & Gerontology International.

[20] Lukacz ES, Santiago-Lastra Y, Albo ME, Brubaker L (2017). Urinary Incontinence in Women: A Review. JAMA.

[21] Leng S, Jin Y, Vitiello MV, Zhang Y, Ren R, Lu L (2023). Self-reported insomnia symptoms are associated with urinary incontinence among older Indian adults: evidence from the Longitudinal Ageing Study in India (LASI). BMC Public Health.

[22] Kwon D, Belsky DW (2021). A toolkit for quantification of biological age from blood chemistry and organ function test data: BioAge. Geroscience.

[23] Gacci M, Sakalis VI, Karavitakis M, Cornu JN, Gratzke C, Herrmann TRW (2022). European Association of Urology Guidelines on Male Urinary Incontinence. Eur Urol.

[24] Herschorn S (2004). Female pelvic floor anatomy: the pelvic floor, supporting structures, and pelvic organs. Rev Urol.

[25] de Groat WC (1997). A neurologic basis for the overactive bladder. Urology.

[26] Bag Soytas R, Soytas M, Danacioglu YO, Citgez S, Yavuzer H, Can G (2021). Relationship between the types of urinary incontinence, handgrip strength, and pelvic floor muscle strength in adult women. Neurourol Urodyn.

[27] Zhu L, Li L, Lang JH, Xu T (2012). Prevalence and risk factors for peri- and postpartum urinary incontinence in primiparous women in China: a prospective longitudinal study. Int Urogynecol J.

[28] Zhang L, Zhu L, Xu T, Lang J, Li Z, Gong J (2015). A Population-based Survey of the Prevalence, Potential Risk Factors, and Symptom-specific Bother of Lower Urinary Tract Symptoms in Adult Chinese Women. Eur Urol.

[29] Cameron AP, Helmuth ME, Smith AR, Lai HH, Amundsen CL, Kirkali Z (2023). Total fluid intake, caffeine, and other bladder irritant avoidance among adults having urinary urgency with and without urgency incontinence: The Symptoms of Lower Urinary Tract Dysfunction Research Network (LURN). Neurourol Urodyn.

[30] Altman D, Granath F, Cnattingius S, Falconer C (2007). Hysterectomy and risk of stress-urinary-incontinence surgery: nationwide cohort study. Lancet.

[31] Mayhew AJ, Amog K, Phillips S, Parise G, McNicholas PD, de Souza RJ (2019). The prevalence of sarcopenia in community-dwelling older adults, an exploration of differences between studies and within definitions: a systematic review and meta-analyses. Age Ageing.

[32] Organization WH (2011). Global health and aging.

[33] Smith L, Sanchez GFL, Veronese N, Soysal P, Kostev K, Jacob L (2022). Association between sarcopenia and quality of life among adults aged ≥ 65 years from low- and middle-income countries. Aging Clin Exp Res.

