# Supplementary material for: The Association Between Sarcopenia and Stress Urinary Incontinence Among Older Adults in India: A Cross-Sectional Study
Source: Int J Med Sci. 2024 Sep 3;21(12):2334–42. doi: 10.7150/ijms.97240 (PMC11413891; doi:10.7150/ijms.97240)
Supplement: Supplementary file 1 — Supplementary table. [file ijmsv21p2334s1.pdf]

Supplementary Table 1. All baseline characteristics of included participants.

Variables	Total	Non-sarcopenia	Sarcopenia	Standardize diff.	P-value
N	42,350	28,068	14,282		
Age (mean ± SD)	62.63 ± 8.98	60.18 ± 7.55	67.45 ± 9.60	0.84 (0.82, 0.86)	<0.001
Waist-hip ratio (mean ± SD)	0.94 ± 0.08	0.94 ± 0.08	0.93 ± 0.08	0.10 (0.08, 0.12)	<0.001
BMI (mean ± SD)	22.59 ± 4.70	23.03 ± 4.57	21.73 ± 4.85	0.28 (0.25, 0.30)	<0.001
BA index (mean ± SD)	0.00 ± 0.11	-0.01 ± 0.11	0.01 ± 0.11	0.18 (0.09, 0.27)	<0.001
Gender, n (%)				0.13 (0.11, 0.15)	<0.001
male	21,450 (50.65%)	14,815 (52.78%)	6,635 (46.46%)		
female	20,900 (49.35%)	13,253 (47.22%)	7,647 (53.54%)		
Education, n (%)				0.31 (0.29, 0.33)	<0.001
never	20,321 (47.98%)	12,159 (43.32%)	8,162 (57.15%)		
middle school or under	14,591 (34.45%)	10,165 (36.22%)	4,426 (30.99%)		
secondary and higher secondary	5,251 (12.40%)	4,004 (14.27%)	1,247 (8.73%)		
above higher secondary	2,187 (5.16%)	1,740 (6.20%)	447 (3.13%)		
Marriage, n (%)				0.38 (0.36, 0.40)	<0.001
Married or partnered	31,233 (73.75%)	22,253 (79.28%)	8,980 (62.88%)		
Widowed	10,152 (23.97%)	5,172 (18.43%)	4,980 (34.87%)		
Others	965 (2.28%)	643 (2.29%)	322 (2.25%)		
Live in a village or a city, n (%)				0.05 (0.03, 0.07)	<0.001
urban	14,513 (34.27%)	9,852 (35.10%)	4,661 (32.64%)		
rural	27,837 (65.73%)	18,216 (64.90%)	9,621 (67.36%)		
Caste, n (%)				0.10 (0.08, 0.12)	<0.001
scheduled caste	70,50 (16.76%)	4,510 (16.19%)	2,540 (17.88%)		
scheduled trible	7,114 (16.91%)	4,813 (17.28%)	2,301 (16.20%)		
other backward class	16,238 (38.61%)	10,461 (37.56%)	5,777 (40.66%)		
no or other caste	11,658 (27.72%)	8,069 (28.97%)	3,589 (25.26%)		
Religion, n (%)				0.07 (0.05, 0.09)	<0.001
Others	2,011 (4.75%)	1,456 (5.19%)	555 (3.89%)		
Hindu	31,367 (74.07%)	20,705 (73.77%)	10,662 (74.65%)		
Muslim	4,871 (11.50%)	3,167 (11.28%)	1,704 (11.93%)		
Christian	4,100 (9.68%)	2,739 (9.76%)	1,361 (9.53%)		
mild physical activity less 1 a week, n (%)				0.24 (0.22, 0.26)	<0.001
0	27,560 (65.14%)	19,348 (68.99%)	8,212 (57.56%)		
1	14,752 (34.86%)	8,696 (31.01%)	6,056 (42.44%)		
vigorous physical activity less 1 a week, n				0.33 (0.31, 0.35)	<0.001

(%)					
0	14,244 (33.67%)	10,862 (38.73%)	3,382 (23.70%)		
1	28,066 (66.33%)	17,180 (61.27%)	10,886 (76.30%)		
Number of CCDs, n (%)				0.15 (0.13, 0.17)	<0.001
0	8,120 (19.29%)	5,750 (20.59%)	2,370 (16.72%)		
1	9,448 (22.44%)	6,479 (23.20%)	2,969 (20.94%)		
2	8,794 (20.89%)	5,885 (21.08%)	2,909 (20.52%)		
3	15,738 (37.38%)	9,809 (35.13%)	5,929 (41.82%)		
Drinking, n (%)				0.07 (0.05, 0.09)	<0.001
never	34,235 (80.92%)	22,430 (80.00%)	11,805 (82.71%)		
ever and current	8,074 (19.08%)	5,607 (20.00%)	2,467 (17.29%)		
Smoking, n (%)				0.03 (0.01, 0.05)	0.013
never	33,654 (79.55%)	22,204 (79.21%)	11,450 (80.23%)		
ever and current	8,650 (20.45%)	5,829 (20.79%)	2,821 (19.77%)		
Care for ADL/IADL, n (%)				0.28 (0.26, 0.30)	<0.001
0	39,476 (93.21%)	26,871 (95.74%)	12,605 (88.26%)		
1	2,874 (6.79%)	1,197 (4.26%)	1,677 (11.74%)		
Stress Urinary Incontinence, n (%)				0.15 (0.13, 0.17)	<0.001
No	38,876 (91.80%)	26,161 (93.21%)	12,715 (89.03%)		
Yes	3,474 (8.20%)	1,907 (6.79%)	1,567 (10.97%)		
BMI categorical, n (%)				0.05 (0.03, 0.07)	<0.001
<30	23,680 (94.28%)	26,034 (92.83%)	13,411 (94.14%)		
>=30	1,436 (5.72%)	2,011 (7.17%)	835 (5.86%)		

Note: BMI, Body mass index; BA index, Biological Aging index; CCDs, Combined Chronic Diseases; ADL, Activities of Daily Living; IADL, Instrumental Activities of Daily Living.
